# Colorectal cancer follow-up after surgical resection since the COVID-19 pandemic: first steps towards out-of-hospital follow-up?

**DOI:** 10.1016/j.esmorw.2024.100070

**Published:** 2024-09-06

**Authors:** H. Swartjes, K.R. Voigt, L. Wullaert, J. Meijer, F.N. van Erning, C. Verhoef, D.J. Grünhagen, P.A.J. Vissers, J.H.W. de Wilt, COVID and Cancer-NL Consortium, S. Siesling, J.C. van Hoeve, M.A.W. Merkx, N.J. de Wit, C.W. Helsper, I. Dingemans, I.D. Nagtegaal, R. Saathof, C.H. van Gils, H.C.P.M. van Weert, M. Verheij

**Affiliations:** 3 Department of Research and Development, Netherlands Comprehensive Cancer Organisation, Utrecht; 4 Department of Health Technology and Services Research, Technical Medical Centre, University of Twente, Enschede; 6 Department of Oral and Maxillofacial Surgery, Radboud University Medical Center, Nijmegen; 7 Department of General Practice, Julius Center for Health Sciences and Primary Care, University Medical Center Utrecht, Utrecht University, Utrecht; 8 Dutch Federation of Cancer Patient Organisations (NFK), Utrecht; 9 Department of Pathology, Radboud University Medical Center, Nijmegen, The Netherlands; 10 On behalf of the Automated Pathology Archive (PALGA); 11 Dutch Hospital Data (DHD), Utrecht; 12 Department of Epidemiology, Julius Center for Health Sciences and Primary Care, University Medical Center Utrecht, Utrecht University, Utrecht; 13 Department of General Practice, Amsterdam Public Health, Amsterdam UMC, Location AMC, Amsterdam; 14 Department of Radiation Oncology, Radboud University Medical Center, Nijmegen, The Netherlands; 15 On behalf of the Dutch Multidisciplinary Oncology Foundation (SONCOS); 1 Department of Surgery, Radboud University Medical Center, Nijmegen; 2 Department of Surgical Oncology and Gastrointestinal Surgery, Erasmus MC Cancer Institute, Rotterdam; 3 Department of Research and Development, Netherlands Comprehensive Cancer Organisation, Utrecht; 4 Department of Health Technology and Services Research, Technical Medical Centre, University of Twente, Enschede; 5 Department of Surgery, Catharina Hospital, Eindhoven, The Netherlands

**Keywords:** aftercare, colorectal neoplasms, follow-up studies, surveillance, telemedicine

## Abstract

**Background:**

The COVID-19 pandemic impacted outpatient clinic services globally. It is unknown how the pandemic affected the follow-up of surgically treated colorectal cancer (CRC) patients. This population-based study aimed to assess the trends in CRC follow-up consultations before and during the COVID-19 pandemic in the Netherlands.

**Materials and methods:**

Nationwide health care activities data between January 2018 and July 2021 were merged with patient-level data from the Netherlands Cancer Registry of stage I-III CRC patients treated with surgical resection. The number of follow-up consultations per patient per year was calculated, and between-group differences were assessed with descriptive statistics. Trends in the number and setting of follow-up consultations were assessed using joinpoint regression analyses. Out-of-hospital follow-up was defined as written, telephone or video consultations.

**Results:**

In total, 42 970 CRC patients were included. The median number of follow-up consultations per year per patient was 2.9 (interquartile range: 2.0-4.7). The median number of follow-up consultations increased with disease stage (*P* < 0.001) and was higher for patients <60 years of age (*P* < 0.001). The total number of follow-up consultations did not change during the study period (*P* = 0.333). The percentage of out-of-hospital follow-up increased from 23% to 80% between January and April 2020 (*P* < 0.001), and remained between 48% and 59% until the end of the study period.

**Conclusions:**

This population-based study showed a great increased use of out-of-hospital consultations during CRC follow-up, which predominantly corresponded to the severity of the COVID-19 pandemic. Future studies should assess whether the use of out-of-hospital follow-up consultations has persisted after the pandemic.

## Highlights

• Out-of-hospital follow-up after treatment of colorectal cancer is relatively new.
• The COVID-19 pandemic has accelerated the development of out-of-hospital follow-up.
• This cohort measured the start of out-of-hospital follow-up in the Netherlands.
• The median number of follow-ups was higher for younger patients.

## Introduction

Colorectal cancer (CRC) is the third most common cancer globally, and the vast majority of patients undergo curative surgical resection.^1^^,^^2^ Subsequently, an oncological follow-up period is initiated to detect potential recurrences in an early stage.^3^ In Western countries, specialist-led follow-up is generally the standard of care, with outpatient visits being the primary setting for these consultations. There is an ongoing debate, however, about the benefit in survival, quality of life, patient-centeredness and cost-effectiveness of specialist-led in-hospital follow-up,^4^, ^5^, ^6^, ^7^, ^8^, ^9^ even before the COVID-19 pandemic was declared by the World Health Organization.^10^

The COVID-19 pandemic heavily impacted outpatient clinic services, and initiated a shift in the setting of follow-up consultations.^11^ Out-of-hospital consultations such as telephone or video consultations were rapidly implemented in care pathways for different oncological diagnoses across the globe.^12^, ^13^, ^14^ In the Netherlands, the Dutch Health Care Authority facilitated this implementation via a regulatory change.^15^ In March 2020, shortly after the first hospital admission with COVID-19 in the nation, the invoicing of out-of-hospital consultations was permitted.

Currently, it is unknown how the COVID-19 pandemic and the subsequent changes in regulation have impacted CRC follow-up on a nationwide basis.^16^ Understanding of recent trends is of importance in the ongoing debate about the most suitable model of follow-up. Moreover, insights in the associations between patient-level factors and follow-up consumption will aid in further personalisation of CRC follow-up. Therefore, the aim of this study was to provide a population-based analysis of trends in CRC follow-up, before and during the COVID-19 pandemic in the Netherlands, with a focus on consultation setting.

## Materials and methods

### Data collection

To enable research into the effects of the COVID-19 pandemic on cancer care, the COVID and Cancer-NL consortium was initiated. This consortium enabled the unique linkage of population-based patient-level tumour, clinical and treatment data from the Netherlands Cancer Registry (NCR) with patient-level health care activity (HCA) declaration data from 69 of 76 (90%) hospitals in the Netherlands from the Dutch National Care Registration (in Dutch: *Landelijke Basisregistratie Ziekenhuizen,* LBZ). The LBZ contains nationwide data of patients who received medical care in a Dutch hospital and is hosted by Dutch Hospital Data (DHD). Publicly available data from the National Institute for Public Health and the Environment (RIVM) were used to describe the number of nationwide COVID-19 hospital admissions.

The NCR is managed by the Netherlands Comprehensive Cancer Organisation (in Dutch: *Integraal Kankercentrum Nederland*, IKNL) and contains the information about all CRC diagnoses since 1989. Dedicated data managers from the NCR extract the data from electronic patient records. Data of patients diagnosed between 1 January 2012 and 31 December 2021 were extracted from the NCR, because these patients could have had follow-up consultations during the study period (i.e. 1 January 2018 to 31 July 2021). Follow-up until vital status was available through linkage with the Dutch Personal Records Database, and was completed until 31 January 2022. DHD routinely collects data on all registered health care activities from hospitals in the Netherlands for the LBZ. Linkage between the NCR and LBZ records was conducted via probabilistic matching on patient level using patient number, date of birth, gender, and postal code.

### Classification of CRC follow-up in this study

The 90th day after the most recent surgical resection of a primary tumour was regarded as the starting point of follow-up. The endpoint of follow-up was the last consultation registered within 5 years plus 90 days after surgical resection, because the recommended duration of CRC follow-up in the Netherlands is 5 years.^3^ The 90-day margin was added to meet slight modifications to the follow-up schedule, which are common in clinical practice. A follow-up consultation was defined as any consultation registered under a CRC diagnosis and which had taken place during the defined follow-up period. Phone, video and written consultations were regarded as out-of-hospital follow-up consultations.

### Patient selection

The study period ranged from 1 January 2018 to 31 July 2021. The start of the study period was defined by the introduction of out-of-hospital consultation registration by the Dutch Health Care Authority on 1 January 2018.^17^ Between 1 January 2018 and 1 March 2020, registration of out-of-hospital consultations was enabled and price agreements could be arranged between hospital and health care insurers. Since 1 March 2020, however, the price of out-of-hospital consultations was equalised with the price of in-hospital follow-up since 1 March 2020. The end of the study period was defined by the period for which HCA declaration data were made available by the COVID and Cancer-NL consortium agreement: 31 July 2021.

Patients aged ≥18 years and diagnosed with stage I-III CRC between 2012 and 2021 were eligible for inclusion; patients diagnosed before 2012 would have finished their 5 years of follow-up before the start of the study period. Patients treated without surgical resection, an appendiceal tumour localisation, or neuroendocrine morphology were excluded from this study. Additionally, duplicate activities on the same day, activities before or after the follow-up period and non-consultation activities were excluded.

Tumour localisation was categorised as either colon (C18.0, C18.2-C18.9) or rectum (C19.9, C20.9). Tumour morphology was coded as non-mucinous adenocarcinoma (8140-8389), mucinous adenocarcinoma (8470, 8480, 8481), signet cell carcinoma (8574) or other. Tumour stage was classified following the eight edition of the Union for International Cancer Control’s Tumour-Node-Metastasis (TNM) Classification of Malignant Tumours.

### Guideline recommendation for CRC follow-up

A guideline revision on the recommended follow-up regimen was presented during the study period.^3^ The prior and present guideline did not provide recommendations on the number of follow-up consultations. In clinical practice, however, each measurement of the carcinoembryonic antigen (CEA) level is combined with a consultation. Before December 2020, one to four CEA measurements were recommended in year 1-3 and one to two measurements in year 4-5. After December 2020, two to four measurements were recommended in year 1-2 only, and one to two measurements were recommended in year 3-5.

### Statistical analyses

#### Patient-level analyses

Patients with less than half a year of follow-up during the study period were excluded from patient-level analyses (*n* = 7242, 16.9%). The number of follow-up consultations per patient per year was calculated within this population (Supplementary Figure S1, available at https://doi.org/10.1016/j.esmorw.2024.100070). For each patient, the number of registered consultations during the study period was divided by the length of follow-up during the study period until the last registered consultation. Between-group differences in the median number of follow-up consultations per year were assessed with the Kruskal–Wallis test.

#### Health care activity-level analyses

The data of all patients (*n* = 42 970) were included in the HCA-level analyses. The total number of follow-up consultations and the percentage of out-of-hospital consultations were calculated per month, and trends in these data were assessed using linear joinpoint regression. Additionally, the ratio between the total number of follow-up consultations and the total number of patients in CRC follow-up during the study period was assessed. Homoscedasticity of the data was assumed. The maximal amount of joinpoints was set at 5, and only the statistically best fitting model based on the weighted Bayesian information criterion was shown.

#### The setting of follow-up consultations

A sub-cohort of patients was created to test for associations between follow-up setting and tumour and patient characteristics. This sub-cohort consisted of consultations during the COVID-19 pandemic only (i.e. since March 2020), to limit the influence of the pre-pandemic period, in which out-of-hospital consultations were uncommon. Patients who had four or more consultations between 1 March 2020 and the end of the study period (i.e. 31 July 2021) were included in this sub-cohort. A cut-off value of ≥75% out-of-hospital consultations of the total number of consultations was used to identify possible associations between patients and tumour characteristics and out-of-hospital follow-up with multivariable logistic regression.

*P* values <0.05 were regarded as statistically significant. Statistical analyses were carried out and plots were created using R version 4.2.2 with the ‘ggplot2’ package, apart from the joinpoint regression analyses (Joinpoint Regression Program, Version 5., April 2023; Statistical Methodology and Applications Branch, Surveillance Research Program, National Cancer Institute).

## Results

**Figure 1:**
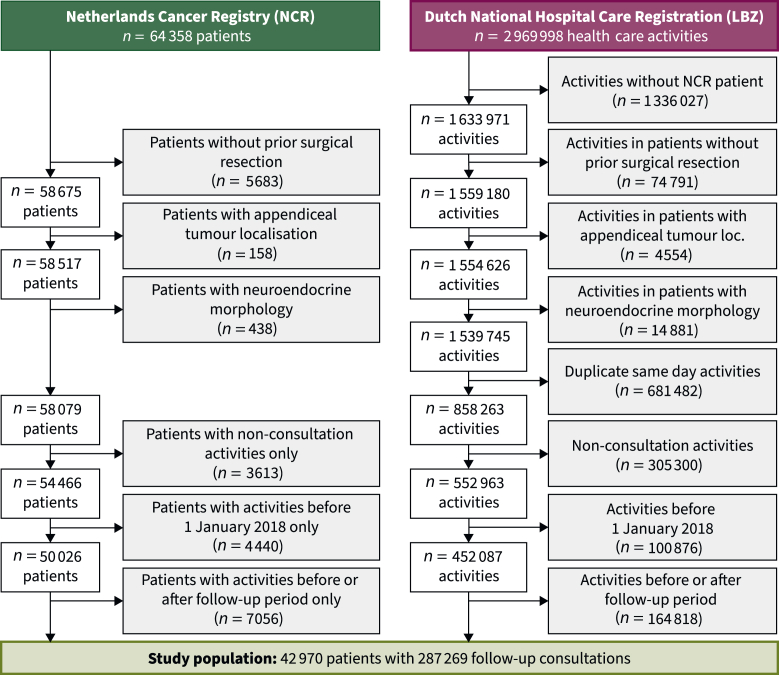
Flowchart of inclusions and exclusions.

This study included 42 970 patients. For these patients, 287 269 CRC follow-up consultations were registered during the study period (Figure 1). An overview of patient and tumour characteristics is available as Table 1.

**Table 1:** **Patient and tumour characteristics (*N* = 42 970).** IQR, interquartile range

	Median (IQR) or *n* (%)
Year of diagnosis	2017 (2015-2019)
Age at diagnosis (years)	69 (61-75)
Sex	
Male	23 846 (55.5)
Female	19 124 (44.5)
Cancer stage	
Stage I	10 241 (23.8)
Stage II	14 473 (33.7)
Stage III	18 256 (42.5)
Localisation	
Colon	30 267 (70.4)
Rectum	12 703 (29.6)
Morphology	
Non-mucinous adenocarcinoma	39 153 (91.1)
Mucinous adenocarcinoma	3558 (8.3)
Signet cell carcinoma	8 (0.0)
Other	251 (0.6)
Type of surgical resection	
Hemicolectomy or ileocecal resection	18 759 (43.7)
Transverse colectomy	622 (1.4)
Sigmoid colectomy	8939 (20.8)
Subtotal colectomy	542 (1.3)
Low anterior resection	10 416 (24.2)
Abdominoperineal resection	3606 (8.4)
Total (procto)colectomy	86 (0.2)
Systemic therapy	
No	28 784 (67.0)
Yes	14 186 (33.0)
Radiation therapy	
No	35 241 (82.0)
Yes	7729 (18.0)
Months of follow-up during study period	20 (9-32)

The median number of follow-up consultations per patient per year was 2.9 (interquartile range: 2.0-4.7). This number increased with disease stage (*P* < 0.001, Table 2). Moreover, stratification for age at diagnosis showed that the age groups <60 years had a higher median number of follow-up consultations (*P* < 0.001). The median differed between patients with rectal and colonic tumours as well, but this difference was small in magnitude (2.9 versus 3.1, *P* < 0.001).

**Table 2:** **Univariable descriptive statistics for the median number of follow-up consultations per patient per year, stratified for patient and tumour characteristics.** The Kruskal–Wallis test was used to test for between-group differences in the median number of follow-up consultations per patient per year

	*n* (%)	Median number of follow-up consultations per patient per year (IQR)	*P* value
Total	35 728 (100%)	2.9 (2.0-4.7)	N/a
Age (years)			**<0.001**
18-49	1719 (4.8)	3.4 (2.2-5.8)	
50-59	5620 (15.7)	3.3 (2.2-5.2)	
60-69	12 012 (33.6)	2.7 (1.9-4.3)	
70-79	12 533 (35.1)	3.0 (2.0-4.7)	
≥80	3844 (10.8)	3.1 (2.1-4.7)	
Sex			0.146
Male	19 912 (55.7)	3.0 (2.0-4.8)	
Female	15 816 (44.3)	2.9 (2.0-4.7)	
Cancer stage			**<0.001**
Stage I	8584 (24.0)	2.5 (1.7-3.7)	
Stage II	12 092 (33.8)	2.8 (2.0-4.2)	
Stage III	15 052 (42.1)	3.5 (2.2-6.1)	
Morphology			0.375
Non-mucinous adenocarcinoma	32 624 (91.3)	2.9 (2.0-4.7)	
Mucinous adenocarcinoma	2905 (8.1)	3.0 (2.0-4.7)	
Signet cell carcinoma	8 (0.0)	4.2 (3.3-7.2)	
Other	191 (0.5)	3.2 (2.0-5.0)	
Localisation			**<0.001**
Colon	24 921 (69.8)	2.9 (2.0-4.6)	
Rectum	10 807 (30.2)	3.1 (2.1-5.0)	

Significant *P*-values are presented in bold.

IQR, interquartile range.

**Figure 2:**
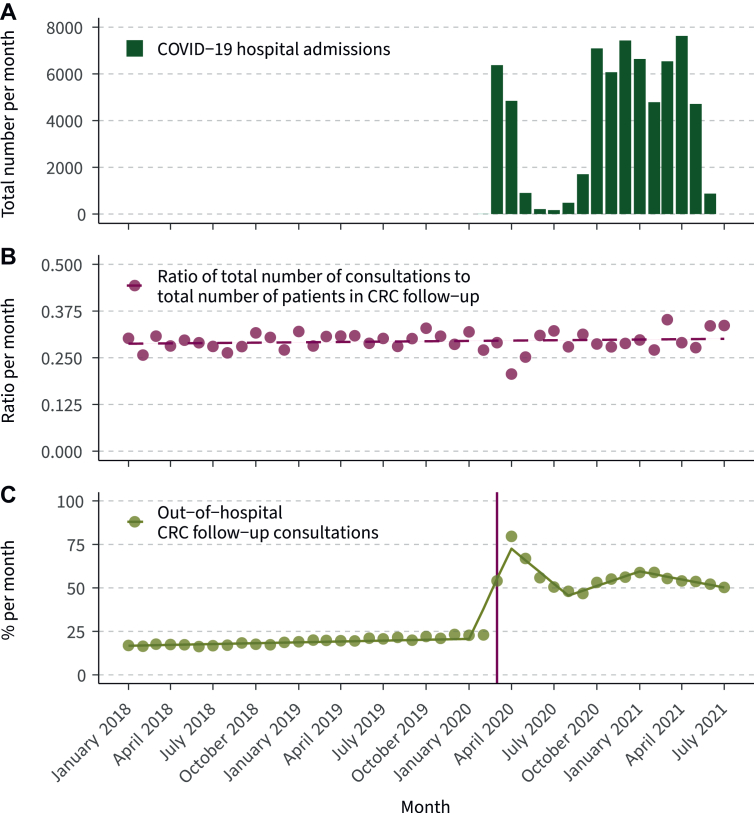
**Trends in COVID-19 hospital admissions, ratio of the total number of follow-up consultations to the total number of patients in CRC follow-up and the percentage of out-of-hospital CRC follow-up consultations per month between January 2018 and July 2021.** The vertical line (C) marks March 2020. In this month, the Dutch Health Care Authority acknowledged the registration and invoicing of out-of-hospital consultations. A dashed line represents a statistically insignificant trend change; a solid line represents a statistically significant trend change. CRC, colorectal cancer.

The first hospital admission for COVID-19 in the Netherlands was registered in February 2020. The number of COVID-19 hospital admissions spiked in March and April 2020, but remained <2000 in the months between May and September 2020 (Figure 2A). The ratio of the total number of follow-up consultations to the total number of patients in follow-up did not change significantly during the study period (*P* = 0.333, Figure 2B). The percentage of out-of-hospital follow-up consultations changed drastically during the study period, however (Figure 2C). A slight increase between January 2018 and January 2020 was noted (16.9% to 22.7%, *P* < 0.001). Between January 2020 and April 2020, the percentage of out-of-hospital follow-up consultations increased extremely from 22.7% to 79.6% (*P* < 0.001). In the period between April 2020 and August 2020, the percentage decreased to 48.0% (*P* < 0.001). This was followed by a slight increase between August 2020 and January 2021 (48.0% to 58.8%, *P* < 0.001), and a slight decrease again between January 2021 and July 2021 (58.8% to 50.3%, *P* < 0.001). The majority of out-of-hospital follow-up consultations were registered as telephone consultations (*N* = 94 694; 99.6%); video (*N* = 214; 0.2%) and written consultations (*N* = 169; 0.18%) were rarely registered.

The sub-cohort analysis showed that an age of ≥80 years [odds ratio (OR): 1.18; 95% confidence interval (CI) 1.01-1.37] and the female sex (OR: 1.14; 95% CI 1.05-1.24) were associated with ≥75% out-of-hospital follow-ups (Table 3). Patients with higher tumour stages (stage II—OR: 0.82; 95% CI 0.73-0.92 and stage III—OR: 0.55; 95% CI 0.49-0.61) or non-radical resections (OR: 0.57; 95% CI 0.57-0.98) were associated with <75% of out-of-hospital consultations.

**Table 3:** **Multivariable logistic regression output of associations with receiving ≥75% out-of-hospital consultations between 1 March 2020 and 31 July 2021.** The model was adjusted for incidence date (OR: 1.0, 95% CI 1.0-1.0) and death during follow-up in study period (OR: 0.27; 95% CI 0.22-0.33)

	*n* (%)	OR (95% CI)	*P* value
Dependent variable			
Percentage of out-of-hospital consultations in total number of follow-up consultations			
<75%	6593 (63.5)		
≥75%	3792 (36.5)	N/a	N/a
**Independent variables**			
Age, years			
18-49	642 (6.2)	1.17 (0.98-1.40)	0.088
50-59	2022 (19.5)	1.01 (0.90-1.14)	0.811
60-69	3100 (29.9)	Reference	
70-79	3599 (34.7)	1.01 (0.91-1.12)	0.822
≥80	1022 (9.8)	1.18 (1.01-1.37)	**0.034**
Sex			
Male	5729 (55.2)	Reference	
Female	4656 (44.8)	1.14 (1.05-1.24)	**0.001**
Cancer stage			
Stage I	1845 (17.8)	Reference	
Stage II	3145 (30.3)	0.82 (0.73-0.92)	**0.001**
Stage III	5395 (51.9)	0.55 (0.49, 0.61)	**<0.001**
Morphology			
Non-mucinous adenocarcinoma	9449 (91.0)	Reference	
Other	936 (9.0)	0.99 (0.86-1.15)	0.921
Resection margin			
R0	9868 (95.0)	Reference	
-2	249 (2.4)	0.57 (0.42-0.78)	**0.001**
Unknown/missing^a^	268 (2.6)	0.75 (0.57, 0.98)	**0.035**
Localisation			
Colon	7110 (68.5)	Reference	
Rectum	3275 (31.5)	0.87 (0.80-0.96)	**0.004**

Significant *P*-values are presented in bold.

CI, confidence interval; IQR, interquartile range; N/a, not applicable; OR, odds ratio; R0, microscopic complete resection margin; R1, microscopic incomplete resection margin; R2, macroscopic incomplete resection margin.

a Category was included to enable for complete case analysis.

## Discussion

This study provided an analysis of unique population-based data about CRC follow-up consultations between January 2018 and July 2021 in the Netherlands. It showed that the median number of follow-up consultations per patient per year was 2.9 (interquartile range: 2.0-4.7) during the study period. The median number of follow-up consultations per year increased with disease stage, and was higher for patients aged <60 years at diagnosis. Moreover, this study showed that the beginning of the COVID-19 pandemic and the subsequent regulatory changes caused a change in the setting of follow-up consultations: from then until the end of the study period, the majority of follow-up consultations were in the out-of-hospital setting. The total number of follow-up consultations did not change significantly during the study period.

According to the recommendations in the guideline of 2014 and 2020, the expected number of follow-up consultations per patient per year would have been between 1.6-3.2 and 1.4-2.8, respectively. The present study showed a median number of 2.9, which is relatively high in comparison with these ranges. This high number of consultations is likely caused by the inclusion of all postoperative consultations associated with a CRC diagnosis at the surgical departments during the follow-up. Additional consultations because of elevated CEA measurements, symptoms or questions related to a CRC diagnosis are therefore included in this number, while consultations related to metastatic disease are not included. The present study herewith provides an actual indication of the number of consultations during follow-up.

A survey study in the Netherlands previously identified, amongst other factors, stage III CRC as associated with more follow-up appointments than recommended.^18^ The results from the present study showed that this association likely holds on the population-level, with a higher number of follow-up consultations for more advanced stages. Age, however, stratified between <60 years and ≥60 years, did not show a significant association in the previous survey study. During the COVID-19 pandemic, CRC patients aged >80 years received more out-of-hospital consultations, possibly due to the higher mortality rates in elderly patients.^19^ It is also possible that follow-up outside the hospital was advantageous for this population due to mobility issues. Additionally, higher disease stages were associated with more in-hospital consultations. No recommendation is provided in the national guideline about variation in intensity of follow-up based on patient or tumour characteristics; only waiving follow-up based on shared decision-making is recommended.^3^ It is, however, reasonable that specific patient and tumour characteristics (e.g. frailty, lower tumour stage, comorbidities) play a role in the choice whether or not to waive CRC follow-up.

Previously conducted studies concluded that the impact of the COVID-19 pandemic on diagnosis and treatment of CRC in the Netherlands was limited,^20^, ^21^, ^22^ but none of these studies mentioned the follow-up period. The present study showed a substantial shift in the setting of follow-up consultations during the pandemic. The percentage of out-of-hospital follow-up consultations increased greatly between January and April 2020 to >80%. Thereafter, this percentage fluctuated between ∼50% and 60%, increasing and decreasing accordingly with the fluctuation in COVID-19 hospital admissions. The authors were unable to identify multicentre or population-based studies which provided percentages of out-of-hospital or telehealth consultations with oncological patients during the COVID-19 pandemic, limiting the comparability of the results. Nonetheless, several quantitative studies described a shift from in-hospital to out-of-hospital consultations with oncological patients during the COVID-19 pandemic,^23^ and evaluated patient satisfaction with out-of-hospital consultations.^12^, ^13^, ^14^^,^^24^, ^25^, ^26^, ^27^, ^28^, ^29^, ^30^ Results differ between the studies, but the general consensus seems that the transition from in-hospital to out-of-hospital consultations was well received by patients and health care providers. Several studies mentioned that the setting should be based on the aim of the consultation,^12^^,^^14^ however, or that patients expressed their explicit preference for in-hospital consultations.^25^^,^^27^^,^^30^

The Dutch Health Care Authority introduced three novel HCAs in January 2018: the screen-to-screen consultation, telephone consultation, and written consultation.^17^ They allowed hospitals and health care insurers to individually negotiate agreements on the price of diagnosis-treatment combinations (i.e. predetermined package of care for a specific diagnosis) for which these HCAs could be used. The results of the present study show that the proportion of out-of-hospital consultations hereafter was relatively low (±20%), suggesting limited use of the introduced HCAs. In March 2020, however, the Dutch Health Care Authority published a statement that the registration of a screen-to-screen, telephone or written consultation would lead to the same price of a diagnosis-treatment combination as registration of an in-hospital face-to-face consultation until further notice.^31^^,^^32^ The primary aim of this regulatory change was to reduce the risk of transmission of COVID-19. The results from the present study show a rapid nationwide implementation of these out-of-hospital consultations. The percentage of out-of-hospital consultations remained above ∼50% for the remainder of the study period. Since then, the Dutch Health Care Authority has implemented the abovementioned regulatory change on a permanent basis,^33^ enabling permanent implementation of out-of-hospital consultations in CRC follow-up.

A systematic review published in 2023 by Xiao et al.^11^ showed that there was enough evidence to conclude that out-of-hospital versus in-hospital follow-up consultations are equally safe in regard to adverse events, recurrence detection and satisfaction for oncology patients. The results from this study will likely aid in the implementation of out-of-hospital consultations for oncological follow-up. Clearly, the COVID-19 pandemic accelerated the development and implementation of alternative models of oncological follow-up.^4^ This was underlined by a recent systematic review from Newton et al., which focused on patient-initiated (or patient-led) follow-up for oncology patients.^34^ The results from the Danish FURCA trial investigation patient-led follow-up for rectal cancer patients fell outside the timeline of the abovementioned systematic review, but showed that this model of follow-up was associated with a decreased number contacts, outpatient clinic visits and equal satisfaction in comparison with standard in-hospital follow-up.^35^ Moreover, quality of life between the two arms was equal.^36^ The results of two ongoing prospective studies in the Netherlands will contribute to the growing body of evidence about the applicability of these alternative models of oncological follow-up.^37^^,^^38^

The present study provided a unique real-world insight into CRC follow-up in the Netherlands, but had several limitations as well. First, no data were available on the endpoint of follow-up for a patient. Reasons for ending CRC follow-up earlier than the recommended 5 years could be diagnosis of a recurrence, emigration abroad, or patient preference. To deal with the unavailability of these data, the last registered CRC-related consultation was taken as the endpoint in the calculation of the number of follow-up consultations per patient per year. Consultations related to metastatic disease or other malignancies were not available, but could have helped to more accurately classify premature ending of the CRC follow-up. Second, apart from the date and setting of the follow-up consultation, no data were available to further classify the consultation. Data about, for example, CEA level or symptoms would have provided a valuable insight into the reason for consultations. Third, no HCA declaration data were available beyond 31 July 2021 due to the end date of the consortium. These data would be valuable to assess the possible persistence of out-of-hospital consultations in CRC follow-up. The present study has shown the applicability of these data for medical research, and the authors would therefore advocate that the availability of HCA declaration data for scientific research purposes will be increased in the future.

This population-based study showed a great increased use of out-of-hospital consultations during CRC follow-up between January 2018 and July 2021, which corresponded to the pressure that COVID-19 hospital admissions put on hospitals. Moreover, the financial compensation for medical specialists for out-of-hospital consultations has been equalised to that of in-hospital consultations since the COVID-19 pandemic, which could promote the persistence of high percentages of out-of-hospital follow-up consultations during CRC follow-up.

## References

[1] Bray F., Ferlay J., Soerjomataram I., Siegel R.L., Torre L.A., Jemal A. (2018). Global cancer statistics 2018: GLOBOCAN estimates of incidence and mortality worldwide for 36 cancers in 185 countries. CA Cancer J Clin.

[2] IKNL Cijfers darmkanker: IKNL. https://iknl.nl/kankersoorten/darmkanker/registratie.

[3] (2020). NVvH. Follow-up bij CRC - Richtlijn - Richtlijnendatabase. https://richtlijnendatabase.nl/richtlijn/colorectaal_carcinoom_crc.

[4] Jefford M., Howell D., Li Q. (2022). Improved models of care for cancer survivors. Lancet.

[5] Qaderi S.M., Swartjes H., Custers J.A.E., de Wilt J.H.W. (2020). Health care provider and patient preparedness for alternative colorectal cancer follow-up; a review. Eur J Surg Oncol.

[6] Augestad K.M., Norum J., Dehof S. (2013). Cost-effectiveness and quality of life in surgeon versus general practitioner-organised colon cancer surveillance: a randomised controlled trial. BMJ Open.

[7] Galjart B., Höppener D.J., Aerts J., Bangma C.H., Verhoef C., Grünhagen D.J. (2022). Follow-up strategy and survival for five common cancers: a meta-analysis. Eur J Cancer.

[8] Stiggelbout A.M., de Haes J.C., Vree R. (1997). Follow-up of colorectal cancer patients: quality of life and attitudes towards follow-up. Br J Cancer.

[9] Wullaert L., Voigt K.R., Verhoef C., Husson O., Grünhagen D.J. (2023). Oncological surgery follow-up and quality of life: meta-analysis. Br J Surg.

[10] Cucinotta D., Vanelli M. (2020). WHO declares COVID-19 a pandemic. Acta Biomed.

[11] Xiao K., Yeung J.C., Bolger J.C. (2023). The safety and acceptability of using telehealth for follow-up of patients following cancer surgery: a systematic review. Eur J Surg Oncol.

[12] Banbury A., Smith A.C., Taylor M.L. (2022). Cancer care and management during COVID-19: a comparison of in-person, video and telephone consultations. J Telemed Telecare.

[13] Street R.L., Treiman K., Kranzler E.C. (2022). Oncology patients’ communication experiences during COVID-19: comparing telehealth consultations to in-person visits. Support Care Cancer.

[14] Leszczynski R., Norori N., Allen S. (2022). Remote consultations: experiences of UK patients with prostate cancer during the COVID-19 pandemic. Future Oncol.

[15] Government of the Netherlands (2020). Government encouraging the use of eHealth (telehealth). https://www.government.nl/topics/ehealth/government-encouraging-use-of-ehealth.

[16] Dupraz J., Le Pogam M.A., Peytremann-Bridevaux I. (2022). Early impact of the COVID-19 pandemic on in-person outpatient care utilisation: a rapid review. BMJ Open.

[17] Zorgautoriteit N. (2018). Informatiekaart consulten op afstand. https://puc.overheid.nl/nza/doc/PUC_297293_22/1/.

[18] Qaderi S.M., Ezendam N.P.M., Verhoeven R.H.A., Custers J.A.E., de Wilt J.H.W., Mols F. (2021). Follow-up practice and healthcare utilisation of colorectal cancer survivors. Eur J Cancer Care (Engl).

[19] Yanez N.D., Weiss N.S., Romand J.-A., Treggiari M.M. (2020). COVID-19 mortality risk for older men and women. BMC Public Health.

[20] Toes-Zoutendijk E., Vink G., Nagtegaal I.D. (2022). Impact of COVID-19 and suspension of colorectal cancer screening on incidence and stage distribution of colorectal cancers in the Netherlands. Eur J Cancer.

[21] Meijer J., Elferink M.A.G., Vink G.R. (2022). Limited impact of the COVID-19 pandemic on colorectal cancer care in the Netherlands in 2020. Int J Colorectal Dis.

[22] Meijer J., Elferink M.A.G., van Hoeve J.C. (2022). Impact of the COVID-19 pandemic on colorectal cancer care in the Netherlands: a population-based study. Clin Colorectal Cancer.

[23] Hui S., Sane N., Wang A. (2023). Hepatocellular carcinoma surveillance in the telehealth era: a single-centre review. J Telemed Telecare.

[24] Farguell J., Holguin V., Gonzalez C. (2022). Telemedicine and intraductal papillary mucinous neoplasms: analysis of a new follow-up strategy during COVID-19 outbreak. Surgery.

[25] Fassas S., Cummings E., Sykes K.J., Bur A.M., Shnayder Y., Kakarala K. (2021). Telemedicine for head and neck cancer surveillance in the COVID-19 era: promise and pitfalls. Head Neck.

[26] Dhillon K., Manji J., Tapia Cespedes M. (2022). Use of telemedicine consultations in head and neck cancer: patient perceptions, acceptability and accessibility. ANZ J Surg.

[27] Turcotte B., Bélanger L., Blais A.S. (2022). Perception and satisfaction of patients after telemedicine urology consultations: a matched analysis with physicians’ perspective. Can Urol Assoc J.

[28] Schuster-Bruce A.T., Middleton H.A.R., Macpherson C., Pearce B.C.S., Evans A. (2021). Patient satisfaction with nurse-led end of treatment telephone consultation for breast cancer during COVID-19 pandemic. Breast J.

[29] Heeno E., Biesenbach I., Englund C., Lund M., Toft A., Lund L. (2021). Patient perspective on telemedicine replacing physical consultations in urology during the COVID-19 lockdown in Denmark. Scand J Urol.

[30] Splinter M.J., Ikram M.K., Helsper C.W., Bindels P.J.E., de Schepper E.I.T., Licher S. (2023). Patient perspectives on telemedicine during the COVID-19 pandemic: a mixed-methods community-based study. BMC Health Serv Res.

[31] Zorgautoriteit N. (2020). Versoepelingen NZa-regels tijdens corona in 2020. https://puc.overheid.nl/nza/doc/PUC_708615_22/1/.

[32] Specialisten F.M. (2020). Verdere versoepeling NZA-regels. https://demedischspecialist.nl/nieuwsoverzicht/nieuws/verdere-versoepeling-nza-regels?page=4.

[33] Zorgautoriteit N. (2023). Wegwijzer bekostiging digitale zorg 2023. https://puc.overheid.nl/nza/doc/PUC_655318_22/1/.

[34] Newton C, Beaver K, Clegg A (2022). Patient initiated follow-up in cancer patients: a systematic review. Front Oncol.

[35] Hovdenak Jakobsen I., Vind Thaysen H., Laurberg S., Johansen C., Juul T., FURCA Steering Group (2021). Patient-led follow-up reduces outpatient doctor visits and improves patient satisfaction. One-year analysis of secondary outcomes in the randomised trial Follow-Up after Rectal CAncer (FURCA). Acta Oncol.

[36] Hovdenak I., Thaysen H.V., Bernstein I.T. (2023). Quality of life and symptom burden after rectal cancer surgery: a randomised controlled trial comparing patient-led versus standard follow-up. J Cancer Surviv.

[37] Swartjes H., Qaderi S.M., Teerenstra S. (2023). Towards patient-led follow-up after curative surgical resection of stage I, II and III colorectal cancer (DISTANCE-trial): a study protocol for a stepped-wedge cluster-randomised trial. BMC Cancer.

[38] Voigt K.R., Wullaert L., Höppener D.J. (2023). Patient-led home-based follow-up after surgery for colorectal cancer: the protocol of the prospective, multicentre FUTURE-primary implementation study. BMJ Open.

