# Supplementary material for: Colorectal cancer follow-up after surgical resection since the COVID-19 pandemic: first steps towards out-of-hospital follow-up?
Source: ESMO Real World Data Digit Oncol. 2024 Sep 6;5:100070. doi: 10.1016/j.esmorw.2024.100070 (PMC12836599; doi:10.1016/j.esmorw.2024.100070)
Supplement: Supplementary Material [file mmc1.docx]

**SUPPLEMENTARY MATERIAL**

**
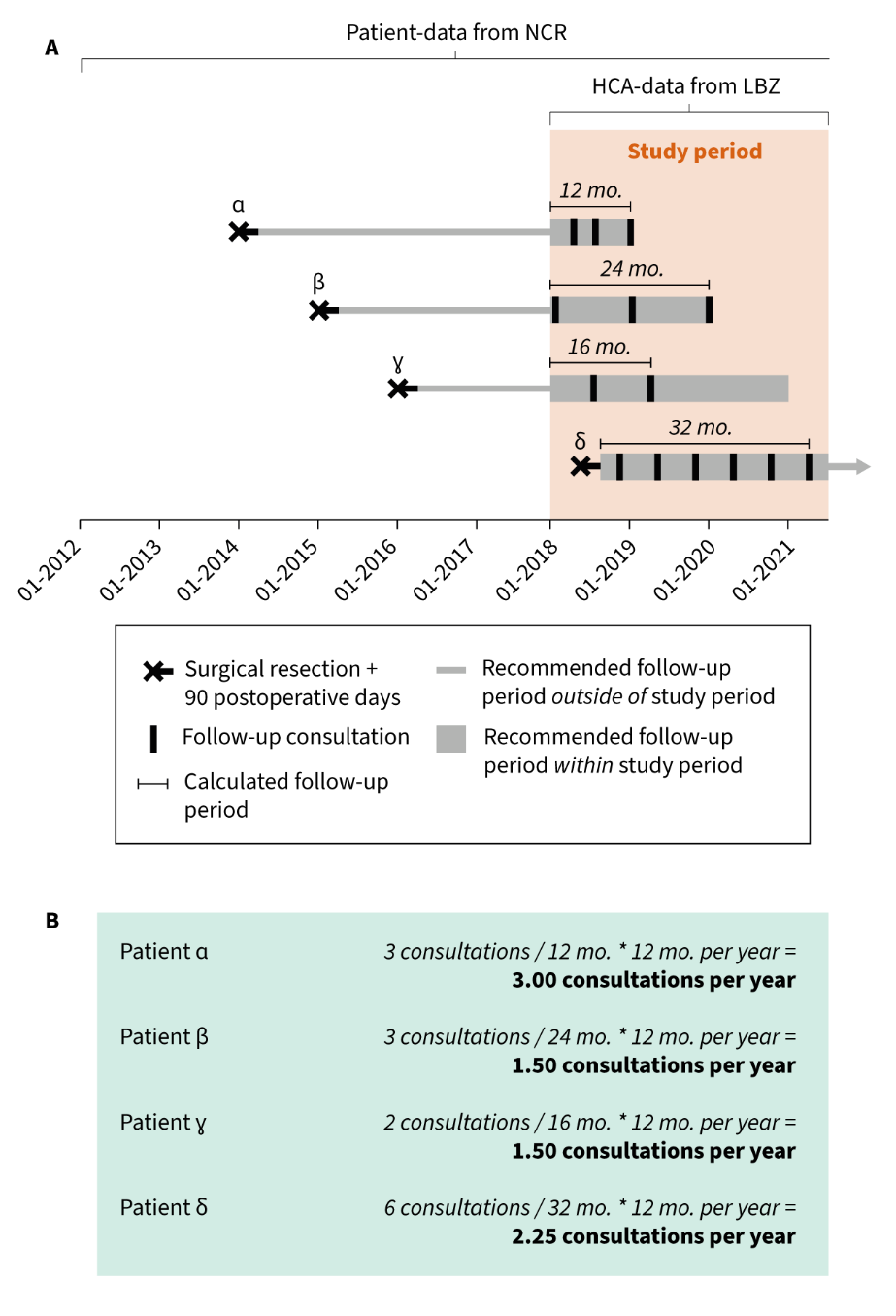
Supplementary Figure 1 –** *Graphical example of the study design (A) and the calculation of the number of follow-up consultations per patients per year (B). NCR; Netherlands Cancer Registry. HCA; Health-care activity. LBZ; Dutch National Hospital Care Registration. Mo; months.*
